# Taurine Inhibits Glucocorticoid-Induced Bone Mitochondrial Injury, Preventing Osteonecrosis in Rabbits and Cultured Osteocytes

**DOI:** 10.3390/ijms21186892

**Published:** 2020-09-20

**Authors:** Hiroaki Hirata, Shusuke Ueda, Toru Ichiseki, Miyako Shimasaki, Yoshimichi Ueda, Ayumi Kaneuji, Norio Kawahara

**Affiliations:** 1 Department of Orthopaedic Surgery, Kanazawa Medical University, Daigaku 1-1, Uchinada-machi, Kahoku-gun, Ishikawa 920-0293, Japan; hiro6246@kanazawa-med.ac.jp (H.H.); adeu221@kanazawa-med.ac.jp (S.U.); orthoped@kanazawa-med.ac.jp (A.K.); kawa@kanazawa-med.ac.jp (N.K.); 2 Department of Pathology 2, Kanazawa Medical University, Daigaku 1-1, Uchinada-machi, Kahoku-gun, Ishikawa 920-0293, Japan; miya0807@kanazawa-med.ac.jp (M.S.); z-ueda@kanazawa-med.ac.jp (Y.U.)

**Keywords:** osteonecrosis, taurine, mitochondrial function, glucocorticoid

## Abstract

Mitochondrial injury has recently been implicated in the pathogenesis of glucocorticoid-induced osteonecrosis. Using cultured osteocytes and a rabbit model, we investigated the possibility that taurine (TAU), which is known to play a role in the preservation of mitochondrial function, might also prevent the development of osteonecrosis. To reduplicate the intraosseous environment seen in glucocorticoid-induced osteonecrosis, dexamethasone (Dex) was added to MLO-Y4 cultured in 1% hypoxia (H-D stress environment). An in vitro study was conducted in which changes in mitochondrial transcription factor A (TFAM), a marker of mitochondrial function, and ATP5A produced by mitochondria, induced by the presence/absence of taurine addition were measured. To confirm the effect of taurine in vivo, 15 Japanese White rabbits were administered methylprednisolone (MP) 20 mg/kg as a single injection into the gluteus muscle (MP+/TAU− group), while for 5 consecutive days from the day of MP administration, taurine 100 mg/kg was administered to 15 animals (MP+/TAU+ group). As a control 15 untreated rabbits were also studied. The rabbits in each of the groups were sacrificed on the 14th day after glucocorticoid administration, and the bilateral femora were harvested. Histopathologically, the incidence of osteonecrosis was quantified immunohistochemically by quantifying TFAM and ATP5A expression. In the rabbits exposed to an H-D stress environment and in MP+/TAU− group, TFAM and ATP5A expression markedly decreased. With addition of taurine in the in vitro and in vivo studies, the expression of TFAM and ATP5A was somewhat decreased as compared with Dex−/hypoxia− or MP−/TAU− group, while improvement was noted as compared with Dex+/hypoxia+ or MP+/TAU− group. In rabbits, the incidence of osteonecrosis was 80% in MP+/TAU− group, in contrast to 20% in the taurine administered group (MP+/TAU+), representing a significant decrease. Since taurine was documented to exert a protective effect on mitochondrial function by inhibiting the mitochondrial dysfunction associated with glucocorticoid administration, we speculated that it might also indirectly help to prevent the development of osteonecrosis in this context. Since taurine is already being used clinically, we considered that its clinical application would also likely be smooth.

## 1. Introduction

Glucocorticoids are excellent therapeutic agents that are used effectively in diverse conditions, notably autoimmune disorders and asthma. The price for this, however, includes many well-known serious side effects including glucocorticoid-induced femoral head osteonecrosis. Glucocorticoid-induced femoral head osteonecrosis occurs at both young and elder ages, and is considered an intractable condition in which destruction of the hip joint markedly impairs quality of life (QOL) by causing pain and impaired ambulation. Once femoral head osteonecrosis is established, surgical intervention such as artificial joint implantation cannot be avoided in most cases. This situation makes the devising of optimal prophylactic countermeasures and greater elucidation of the underlying pathogenetic mechanisms of glucocorticoid-related injury very important so as to make glucocorticoid use safer.

However, despite the extensive research being focused on glucocorticoid-induced femoral head osteonecrosis, its causes and pathophysiology are still far from clear. Various studies using rabbit, rat, and other animal models have been conducted, with models of rabbit osteonecrosis induced by glucocorticoid administration being the most common [1]. Hitherto, various causative factors such as oxidative stress, vascular endothelium injury, coagulopathy, and dyslipidemia have been implicated, and much work has been devoted to their prevention and management [2,3,4]. Recently, attention has been turned to mitochondria because they are the site of oxidative stress development, and the involvement of mitochondrial injury in glucocorticoid-induced osteonecrosis is being recognized [5].

In general, cells of the mitochondrial electron transport system in vivo account for ≥90% of oxygen consumption, of which 1–5% is converted into reactive oxygen species, representing the major intracellular source of reactive oxygen species generation [6]. The acceleration of reactive oxygen species production induced by mitochondrial and mitochondrial DNA injury has been shown to be involved in the development of diverse pathological conditions. Moreover, it has been recognized that mitochondria exist in a state characterized by constant exposure to oxidative stress, with this having a considerable impact on disease development and progression. For this reason, the possibility has been raised that mitochondria may be an ideal target for both therapeutic trials and attempts at elucidating the underlying pathogenetic mechanisms of various disorders [7].

Taurine (TAU, 2-aminoethanesulfonic acid) has been attracting increasing attention as an easy to administer therapeutic agent with few side effects that can help to prevent mitochondrial injury. Taurine is made up of free amino acids present in large quantities in vivo, and has been proven to be effective in the therapy of mitochondrial cytopathies such as mitochondrial myopathy, encephalopathy, lactic acidosis, stroke-like episodes (MELAS) syndrome [8]. Taurine’s anti-inflammatory and anti-oxidative actions have been exciting interest [9,10], while its impact on mitochondrial function including regenerative and prophylactic actions is also being reported [11,12].

These properties of taurine suggest to us the realistic possibility that it may also be effective in preserving mitochondrial function and thereby help to inhibit the development of glucocorticoid-induced osteonecrosis in which mitochondrial dysfunction has been implicated. In a recent study conducted under in vitro conditions, osteocytes exposed to a stress environment (H-D stress environment), namely a hypoxic environment to which dexamethasone (Dex) had been added, an intraosseous environment with successful reduplication of glucocorticoid-induced osteonecrosis has been described [13]. Here, in an in vitro study, we first sought to determine whether taurine exerts any inhibitory effect on osteocyte mitochondrial functional injury in this kind of H-D stress environment. Furthermore, to document any in vivo inhibitory effect of taurine on the development of glucocorticoid-induced osteonecrosis, we investigated the incidence of osteonecrosis and intraosseous mitochondrial function using a glucocorticoid-induced rabbit osteonecrosis model.

## 2. Results

### 2.1. TFAM and ATP5A Expression Due to Taurine Addition yo Cultured Osteocytes in an H-D Stress Environment

**Figure 1:**
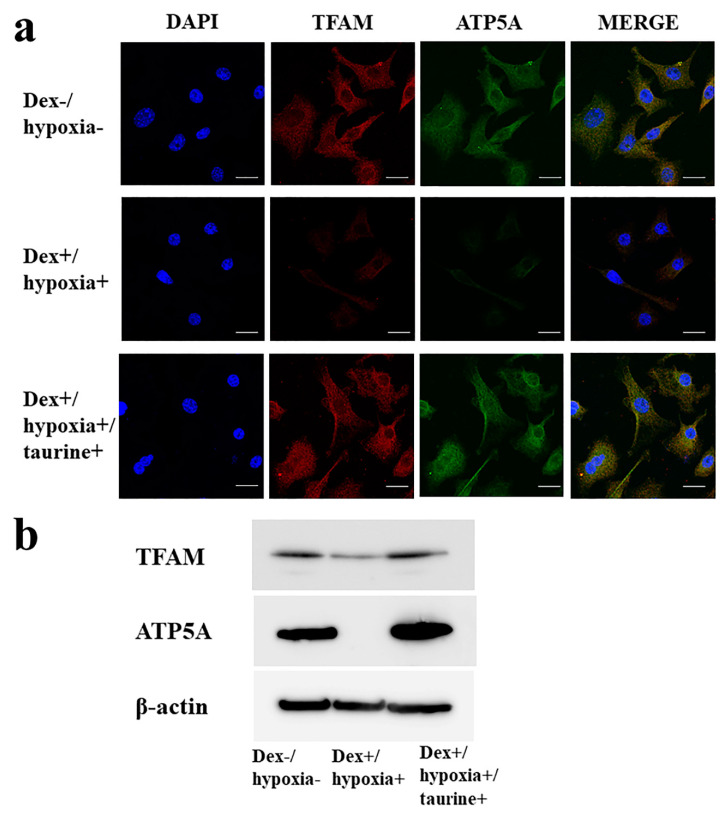
Mitochondrial transcription factor A (TFAM) and ATP5A expression in cultured osteocytes in an H-D stress environment. (**a**) Immunocytochemical study, (**b**) Western blot. TFAM (29 kDa), ATP5A (50–55 kDa), β-actin (42 kDa). In Dex+/hypoxia+ group as compared with Dex−/hypoxia− group, expression of TFAM and ATP5A levels was decreased. In Dex+/hypoxia+/taurine+ group with addition of taurine, as compared with Dex+/hypoxia+ group, levels of both TFAM and ATP5A expression were increased. Scale bar: 20 µm.

To confirm the functional preservation effect of mitochondria due to taurine, the expression of TFAM and ATP5A in osteocytes placed in an H-D stress environment was investigated. In the Dex+/hypoxia+ group, as compared with Dex−/hypoxia− group as a control, the expression of both TFAM and ATP5A decreased. In contrast, in the taurine addition group, the expression of both TFAM and ATP5A in Dex+/hypoxia+/taurine+ group as compared with Dex+/hypoxia+ group was markedly increased (Figure 1). In this way, it was confirmed that taurine preserved mitochondrial function in a stress environment.

### 2.2. Prevention of Osteonecrosis by Taurine in a Glucocorticoid-Administered Rabbit Osteonecrosis Model

**Figure 2:**
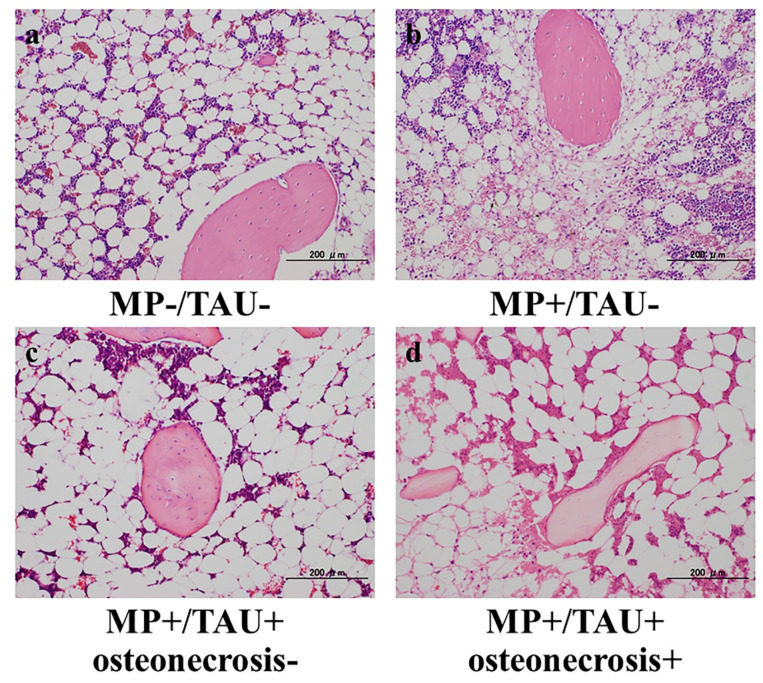
Osteonecrosis inhibition with intravenous administration of taurine in a glucocorticoid-induced rabbit osteonecrosis model. (**a**) MP−/TAU−, (**b**) MP+/TAU−, (**c**,**d**) MP+/TAU+. Osteonecrosis was found in 12 of 15 rabbits of MP+/TAU− group (**b**). In 4 of 15 rabbits of the MP+/TAU+ group, some portions showing necrosis of medullary haematopoietic cells or fat cells and empty lacunae or condensed nuclei in osteocytes were found (**d**). The osteonecrosis development rate was 20%, representing a significant inhibition of osteonecrosis as compared with the MP+/TAU− group (*p* < 0.05). Scale bar: 200 µm.

After preparing a glucocorticoid-administered rabbit osteonecrosis model, taurine was injected intravenously, and its inhibitory effect on osteonecrosis development was determined. In none of the animals in MP−/TAU− group, were there any sites showing necrosis of medullary haematopoietic cells or fat cells, and no empty lacunae or condensed nuclei in osteocytes were found. In 12 of 15 rabbits in the MP+/TAU− group, osteonecrosis was found. In 4 of 15 rabbits in the MP+/TAU+ group, necrosis of medullary haematopoietic cells or fat cells and empty lacunae or condensed nuclei in osteocytes were noted at some sites, and the osteonecrosis development rate was 20% (*p* < 0.05 vs. MP+/TAU−) (Figure 2). Since the decrease in the osteonecrosis development rate was significant, taurine was suggested to have inhibited osteonecrosis development in this experiment.

### 2.3. Inhibition of Mitochondrial Injury by Taurine in a Glucocorticoid-Administered Rabbit Osteonecrosis Model

**Figure 3:**
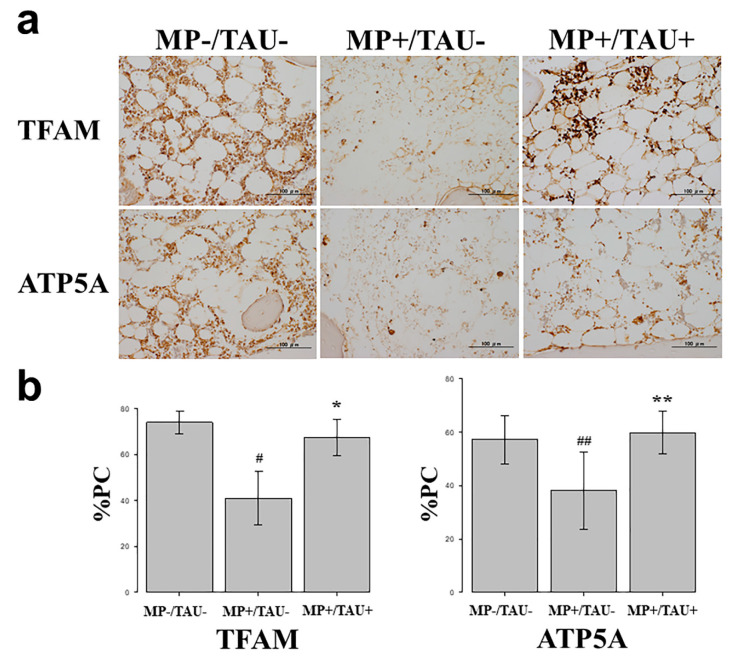
TFAM and ATP5A positive cell rate after taurine administration in a glucocorticoid-induced rabbit osteonecrosis model. (**a**) Immunohistochemical study of TFAM and ATP5A. Scale bar: 100 µm. (**b**) Graph indicates percentage of positive cells of TFAM and ATP5A in the indicated conditions. Columns and bars indicate means and S.E. As compared with MP−/TAU− group, in MP+/TAU− group the numbers of TFAM and ATP5A positive cells were significantly decreased (^#^ *p* < 0.0001 vs. MP−/TAU−, ^##^ *p* = 0.001 vs. MP−/TAU−). In MP+/TAU+ group, TFAM and ATP5A levels were maintained as compared with MP−/TAU− group. (* *p* < 0.0001 vs. MP+/TAU−, ** *p* < 0.001 vs. MP+/TAU−).

To determine any mitochondrial protective effect of taurine in a glucocorticoid-administered rabbit osteonecrosis model, using the percentage of positive cells (%PC) in TFAM and ATP5A, the inhibitory effect of taurine on intraosseous mitochondria injury was investigated. In the MP−/TAU− group, TFAM was 73.95 ± 1.20% and ATP5A was 57.22 ± 2.23%. In the MP+/TAU− group, TFAM was 41.15 ± 2.93%, and ATP5A 31.85 ± 3.61% in contrast to MP+/TAU+ group in which TFAM was 67.19 ± 1.57%, and ATP5A 59.71 ± 2.01%, with increased expression found in this order (Figure 3). In this way, the preservation by taurine of mitochondrial function could be confirmed also in vivo.

## 3. Discussion

Recently, numerous studies have identified a role for oxidative stress in glucocorticoid-induced osteonecrosis [2,14,15]. Regarding mitochondria, which are the major site of oxidative stress generation, the intraosseous decrease of their TFAM levels induced by glucocorticoid administration has been implicated in osteonecrosis development, suggesting that mitochondrial stress plays a role in this process [5]. Accordingly, preservation of mitochondrial function may be an important factor helping to prevent osteonecrosis development [16].

The taurine used in this experiment has lately been recognized to be a useful therapeutic agent in various disorders, including cerebrovascular injury, cardiac disorders, and hepatic injury, by protecting cells from oxidative stress. Other beneficial effects lately emphasized include inhibition of cell injury and anti-ageing properties [9,12,17]. Taurine helps to keep Ca^2+^ concentrations in cardiomyocyte mitochondria normal, even when they are increased by H_2_O_2_-induced oxidative stress, and a possible action on the apoptosis pathway of mitochondria exposed to oxidative stress has been proposed [17]. Moreover, in mitochondrial cytopathies such as MELAS, taurine has attracted attention as a therapeutic agent that provides symptomatic improvement [8]. These findings allow hope that taurine may also help to inhibit mitochondrial injury, which is now thought to be an important step in the course of development of glucocorticoid-induced osteonecrosis.

In this study, to evaluate the efficacy of taurine against mitochondrial injury present in bone and osteocytes, TFAM, a factor strongly involved in mitochondrial function [5,16,18,19] and ATP5A, a marker of mitochondrial function produced by mitochondria, were used.

First, in vitro studies using cultured osteocytes were conducted. In these osteocytes exposed to a sufficiently stressful H-D environment so as to induce cell necrosis, both TFAM and ATP5A expression levels were markedly decreased, proving that mitochondrial functional injury had been engendered. On the other hand, with the addition of taurine under the same conditions, the decrease in TFAM and ATP5A levels was attenuated. These results show that taurine is firmly protective of mitochondrial function in vulnerable osteocytes exposed to a stressful environment.

In various glucocorticoid-induced osteonecrosis models, the occurrence of oxidative and mitochondrial injuries within 48–72 h after glucocorticoid administration is considered to be a contributing factor [2,5,15]. The period from injury to osteonecrosis development has been estimated to be approximately 72–120 h [15,20,21]. Referring to these estimates, in the present glucocorticoid-induced rabbit osteonecrosis model, taurine was administered for 5 consecutive days, and its dose was set to be consistent with that routinely used clinically, 100 mg/kg/day. With glucocorticoid-alone administration (MP+/TAU− group), the intraosseous expression of TFAM and ATP5A was significantly decreased as compared with the MP−/TAU− group. On the other hand, in the MP+/TAU+ group, similar to the in vitro results, TFAM and ATP5A showed improvement up to the levels seen in MP−/TAU− group, as well as a significant decrease in the incidence of osteonecrosis. In this way, together with the mitochondrial injury associated with glucocorticoid use leading to osteonecrosis development, it was considered that taurine protected or preserved mitochondrial function, thereby in turn, possibly helping to prevent the development of osteonecrosis.

For the purposes of the present work, the taurine dose was set to be comparable to that routinely used in daily clinical practice, but because side effects are few, in conditions such as MELAS even larger doses are being administered. In this study, although the osteonecrosis incidence was 20%, to be able to achieve an incidence approaching 0%, factors such as the administered dose of taurine, and its timing and duration will need to be determined. Since a significant decrease in the incidence of glucocorticoid-induced osteonecrosis was attained, considering the ease of taurine administration and the paucity of associated side effects, its clinical application in the near future can be anticipated.

In conclusion, preservation of intraosseous mitochondrial function is vital for the prevention of glucocorticoid-induced osteonecrosis. From the in vivo and in vitro results of taurine obtained here, preservation of mitochondrial function was demonstrated, and was in turn, considered to be related to the prevention of osteonecrosis.

## 4. Materials and Methods

### 4.1. Cell Culture

An established murine osteocytic cell line (MLO-Y4) (Kerafast, Boston, MA, USA) was maintained as a subconfluent monolayer culture in the alpha MEM medium (Gibco, Tokyo, Japan) supplemented with 10% fetal calf serum. When the culture reached 70% confluency being cultured at 37 °C under 20% O_2_ and 5% CO_2_, MLO-Y4 were treated with 1 µM dexamethasone (Dex, MSD, Tokyo, Japan) in 1% O_2_ (hypoxia) for 24 h (Dex+/hypoxia+ group). A quantity of 0.8 µM taurine was added to the medium of Dex+/hypoxia+ group exposed to taurine-free medium (Dex+/hypoxia+/taurine+ group). As a control group, cells were cultured under 20% O_2_ in the culture medium without either Dex or taurine (Dex-/hypoxia− group). Three independent experiments each were carried out.

### 4.2. Immunocytochemical Study

To determine the effect of taurine on cultured osteocytes in an H-D stress environment, an immunohistochemical study on TFAM which exerts a protective effect on mitochondria, and ATP5A which shows ATP production, which is the main function of mitochondria was conducted. Cells of each group were fixed in 4% paraformaldehyde, and permeabilized with 0.3% Triton X-100 in phosphate buffered saline (PBS). Blocking was then achieved with 10% bovine serum albumin (Dako Cytomation, Santa Clara, CA, USA) in PBS, and the primary antibody, anti-ATP synthase (ATP5A) (Proteintech, Rosemont, IL), and anti-TFAM (LSbio, Seattle, MA, USA) antibody each at 10.0 µg/mL were made to react at room temperature for 2 h. The secondary antibody, anti-mouse Alexa 488 (Thermo Fisher Scientific, Tokyo, Japan), and anti-rabbit Alexa 594 (Thermo Fisher Scientific) each at 10.0µg/mL were made to react while being shielded from light, and nucleus staining was done with DAPI. Then after washing in PBS, they were mounted using a prolong diamond antifade mountant (Thermo Fisher Scientific, Tokyo, Japan). Images were taken with a Zeiss-LSM710 camera (Zeiss, Baden-Württemberg, Germany). The taurine non-administered and administered groups were then compared.

### 4.3. Western Blot

For quantification, immunoblotting for TFAM and ATP5A was performed on MLO-Y4 cells. Protein was extracted using protein extraction solution (PRO-PREP, iNtRON Biotechnology, Kyungki-Do, Korea). The protein, 20 µg, was electrophoresed on a 10% polyacrylamide gel, and transferred to a nitrocellulose membrane (Atoh, Tokyo, Japan). The membranes were reacted overnight at 4 ˚C with the primary antibodies. The Primary antibodies applied were anti-ATP5A (Proteintech) or anti-TFAM (Invitrogen, Waltham, MA, USA) antibody at a concentration of 0.5 µg/mL. After the incubation with peroxidase-labeled goat anti-mouse or anti-rabbit IgG antibody (Dako Cytomation, Santa Clara, CA, USA) at a concentration of 0.7 µg/mL each for 1 h at room temperature and vigorous washing, the nitrocellulose membrane was incubated with Chemiluminescence Luminol Reagent (Immuno Star LD, Wako, Tokyo, Japan) and photographed digitally using ImageQuant LAS 4000 mini (GE healthcare Japan Co, Tokyo, Japan). Immunoblot using anti-actin monoclonal antibody (Sigma Chemical Co. St. Louis, MO, USA) was used for standardization. Intensity was measured using the Multi Gauge v3.1 (Fujifilm, Tokyo, Japan). Experiments were repeated at least three times.

### 4.4. Animals

As the experimental animals, forty-five male Japanese White rabbits weighing about 3.5 kg each were used. As the glucocorticoid-induced rabbit osteonecrosis model [1], methylprednisolone (MP) 20 mg/kg was injected into a gluteus muscle only once in 15 rabbits of MP+/TAU− group. In 15 animals of MP+/TAU+ group, after MP injection into a gluteus muscle, taurine 100 mg/kg was injected via an auricular vein for 5 continuous days. A control group was also prepared in which physiological saline 3 mL was injected into a gluteus muscle in 15 rabbits (MP−/TAU− group). The animals in all of the groups were sacrificed 2 weeks later with thiopental sodium (NIPRO ES Pharma, Osaka, Japan) delivered by rapid injection via an auricular vein. The bilateral femora were then quickly resected. After fixation of the resected femurs in 10% neutral buffered formalin, decalcification was done with formic acid, and paraffin embedded specimens were cut into 4 μm thick sections with a microtome. This study was conducted in accordance with all guidelines of the Animal Research Committee of Kanazawa Medical University (#2018-13; approval date: 1 April 2020).

### 4.5. Histopathology

From the femoral sections of each group, sections thinly cut with a microtome from the femoral neck maximum cut surface of the forehead surface were used as hematoxylin and eosin (H&E)-stained specimens, and the presence/absence of osteonecrosis development was studied using a light microscope. Osteonecrosis was judged to be present when necrosis of medullary haematopoietic cells or fat cells or empty lacunae or condensed nuclei in osteocytes were noted [1]. Regarding the osteonecrosis development rate, all of the groups were compared with osteonecrosis considered to be present even if limited to a unilateral leg.

### 4.6. Immunohistochemical Study

To study the effect of taurine in a glucocorticoid-induced rabbit osteonecrosis model in vitro, an immunohistochemical study was performed using TFAM which has a mitochondria protective effect and ATP5A which shows ATP production. In all groups, femoral sections were prepared, and sections obtained from the femoral proximal medial diaphysis of each group were deparaffinized with xylene and ethanol, after which to activate antigen, protease K (Dako Cytomation, Santa Clara, CA, USA) was dribbled on tissue sections that were incubated at 37 °C for 40 min. Using 0.3% H_2_O_2_, endogenous peroxidase was eliminated, blocking was performed using mouse or goat normal serum, and the primary antibody was made to react. As the primary antibody, anti-TFAM rabbit polyclonal antibody (Invitrogen, Waltham, MA, USA) and anti-ATP5A goat polyclonal antibody (Lsbio, Seattle, MA, USA) were used at 0.1 µg/mL and 5.0 µg/mL, respectively. The reaction time was overnight at 4 °C in a cool dark room. After reacting the primary antibody, the secondary antibody (biotin), was reacted with an enzymatic agent (streptavidin), and after 5-minue immersion in DAB to allow for color development, the nuclei were stained and studied under a light microscope.

To quantify relative stainability, 3 fields were chosen at random from tissue adjacent to areas of osteonecrosis in the femoral proximal medial diaphysis, and the proportion of the positive cell count relative to that of the total cell number was calculated, and compared between each group as the percentage of positive cells (%PC).

### 4.7. Statistical Analysis

All quantified results were expressed as the mean ± SE. Significant differences in the osteonecrosis development rate in the H&E-stained specimens, were determined by Fisher’s exact test. Statistical significance in the comparison of %PC of TFAM or ATP5A between the control and each of the experimental groups was analyzed with Dunnett’s multiple comparison test. Significance was defined as *p* < 0.05. The statistical analysis was performed using Stat View J-5.0 software (SAS Institute, Cary, USA)

## Abbreviations

H-D stress environment	dexamethasone was added to MLO-Y4 cultured in 1% hypoxia
TFAM	mitochondrial transcription factor A
Dex	dexamethasone
MP	methylprednisolone
TAU	taurine
QOL	quality of life
MELAS	mitochondrial myopathy, encephalopathy, lactic acidosis, stroke-like episodes
H&E-stain	hematoxylin and eosin-stain
%PC	percentage of positive cells

## References

[1] Yamamoto T., Irisa T., Sugioka Y., Sueishi K. (1997). Effects of pulse methylprednisolone on bone and marrow tissues: Corticosteroid-induced osteonecrosis in rabbits. Artritis Rheum..

[2] Ichiseki T., Matsumoto T., Nishino M., Kaneuji A., Katsuda S. (2004). Oxidative stress and vascular permeability in steroid-induced osteonecrosis model. J. Orthop. Sci..

[3] Kuroda Y., Akiyama H., Kawanabe K., Tabata Y., Nakamura T. (2010). Treatment of experimental osteonecrosis of the hip in adult rabbits with a single local injection of recombinant human FGF-2 microspheres. J. Bone Miner. Metab..

[4] Motomura G., Yamamoto T., Miyanishi K., Jingushi S., Iwamoto Y. (2004). Combined effects of an anticoagulant and a lipid-lowering agent on the prevention of steroid-induced osteonecrosis in rabbits. Arthritis Rheum..

[5] Tsuchiya M., Ichiseki T., Ueda S., Ueda Y., Shimazaki M., Kaneuji A., Kawahara N. (2018). Mitochondrial stress and redox failure in steroid-associated osteonecrosis. Int. J. Med. Sci..

[6] Papa S. (1996). Mitochondrial oxidative phosphorylation changes in the life span. Molecular aspects and physiopathological implications. Biochim. Biophys. Acta..

[7] Oyewole A.O., Birch-Machin M.A. (2015). Mitochondria-targeted antioxidants. FASEB J..

[8] Fakruddin M., Wei F.Y., Suzuki T., Asano K., Kaieda T., Omori A., Izumi R., Fujimura A., Kaitsuka T., Miyata K. (2018). Defective Mitochondrial tRNA Taurine Modification Activates Global Proteostress and Leads to Mitochondrial Disease. Cell. Rep..

[9] Liu Y., Li F., Zhang L., Wu J., Wang Y., Yu H. (2017). Taurine alleviates lipopolysaccharide-induced liver injury by anti-inflammation and antioxidants in rats. Mol. Med. Rep..

[10] Cheleschi S., De-Palma A., Pascarelli N.A., Giordano N., Galeazzi M., Tenti S., Fioravanti A. (2017). Could Oxidative Stress Regulate the Expression of MicroRNA-146a and MicroRNA-34a in Human Osteoarthritic Chondrocyte Cultures?. Int. J. Mol. Sci..

[11] Ahmadi N., Ghanbarinejad V., Ommati M.M., Jamshidzadeh A., Heidari R. (2018). Taurine prevents mitochondrial membrane permeabilization and swelling upon interaction with manganese: Implication in the treatment of cirrhosis-associated central nervous system complications. J. Biochem. Mol. Toxicol..

[12] Wang Q., Fan W., Cai Y., Wu Q., Mo L., Huang Z., Huang H. (2016). Protective effects of taurine n traumatic brain injury via mitochondria and cerebral blood flow. Amino Acids.

[13] Ueda S., Ichiseki T., Yoshitomi Y., Yonekura H., Ueda Y., Kaneuji A., Matsumoto T. (2015). Osteocytic cell necrosis is caused by a combination of glucocorticoid-induced Dickkopf-1 and hypoxia. Med. Mol. Morphol..

[14] Ichiseki T., Kaneuji A., Katsuda S., Ueda Y., Sugimori T., Matsumoto T. (2005). DNA oxidation injury in bone early after steroid administration is involved in the pathogenesis of steroid-induced osteonecrosis. Rheumatology (Oxford).

[15] Ichiseki T., Kaneuji A., Ueda Y., Nakagawa S., Mikami T., Fukui K., Matsumoto T. (2011). Osteonecrosis development in a novel rat model characterized by a single application of oxidative stress. Arthritis Rheum..

[16] Ueda S., Shimasaki M., Ichiseki T., Hirata H., Kawahara N., Ueda Y. (2020). Mitochondrial Transcription Factor an Added to Osteocytes in a Stressed Environment Has a Cytoprotective Effect. Int. J. Med. Sci..

[17] Wang J., Qi C., Liu L., Zhao L., Cui W., Tian Y., Liu B., Li J. (2018). Taurine Protects Primary Neonatal Cardiomyocytes Against Apoptosis Induced by Hydrogen Peroxide. Int. Heart J..

[18] Alam T.I., Kanki T., Muta T., Ukaji K., Abe Y., Nakayama H., Takio K., Hamasaki N., Kang D. (2003). Human mitochondrial DNA is packaged with TFAM. Nucleic Acids Res..

[19] Kanki T., Ohgaki K., Gaspari M., Gustafsson C.M., Fukuoh A., Sasaki N., Hamasaki N., Kang D. (2004). Architectural role of mitochondrial transcription factor A in maintenance of human mitochondrial DNA. Mol. Cell Biol..

[20] Sato M., Sugano N., Ohzono K., Nomura S., Kitamura Y., Tsukamoto Y., Ogawa S. (2001). Apoptosis and expression of stress protein (ORP150, HO1) during development of ischaemic osteonecrosis in the rat. J. Bone Joint Surg. Br..

[21] Catto M. (1965). A histological study of avascular necrosis of the femoral head after transcervical fracture. J. Bone Joint Surg. Br..

